# Tumor-initiating cell frequency is relevant for glioblastoma aggressiveness

**DOI:** 10.18632/oncotarget.11600

**Published:** 2016-08-25

**Authors:** Cristina Richichi, Daniela Osti, Massimiliano Del Bene, Lorenzo Fornasari, Monica Patanè, Bianca Pollo, Francesco DiMeco, Giuliana Pelicci

**Affiliations:** ^1^ Department of Experimental Oncology, European Institute of Oncology, 20139, Milan, Italy; ^2^ Department of Neurosurgery, IRCCS Foundation Neurological Institute “C. Besta”, 20133, Milan, Italy; ^3^ Department of Neuropathology, IRCCS Foundation Neurological Institute “C. Besta”, 20133, Milan, Italy; ^4^ Department of Neurosurgery, Johns Hopkins University, Baltimore, MD 21218, USA; ^5^ Department of Translational Medicine, Piemonte Orientale University “Amedeo Avogadro”, 28100 Novara, Italy

**Keywords:** glioblastoma, tumor-initiating cell frequency, neurosphere, limiting dilution assay, tumorigenicity

## Abstract

Glioblastoma (GBM) is maintained by a small subpopulation of tumor-initiating cells (TICs). The arduous assessment of TIC frequencies challenges the prognostic role of TICs in predicting the clinical outcome in GBM patients. We estimated the TIC frequency in human GBM injecting intracerebrally in mice dissociated cells without any passage in culture.

All GBMs contained rare TICsand were tumorigenic *in vivo* but only 54% of them grew *in vitro* as neurospheres. We demonstrated that neurosphere formation *in vitro* did not foretell tumorigenic ability *in vivo* and frequencies calculated *in vitro* overestimated the TIC content.

Our findings assert the pathological significance of GBM TICs. TIC number correlated positively with tumor incidence and inversely with survival of tumor-bearing mice. Stratification of GBM patients according to TIC content revealed that patients with low TIC frequency experienced a trend towards a longer progression free survival. The expression of either putative stem-cell markers or markers associated with different GBM molecular subtypes did not associate with either TIC content or neurosphere formation underlying the limitations of TIC identification based on the expression of some putative stem cell-markers.

## INTRODUCTION

Although the past years have witnessed an improvement in the understanding of early molecular events in malignant primary CNS tumors, and a plethora of new therapies targeting these events are now tested in clinical trials, effective treatments for most primary malignant CNS tumors are still lacking [1]. Among high-grade gliomas, glioblastoma (GBM; World Health Organization grade IV astrocytoma) [2] is the most aggressive primary tumor in adults with dismal prognosis. Its treatment is palliative and includes surgery, radiotherapy, and concomitant chemotherapy [3].

Tumor heterogeneity depicts the leading feature supporting tumor robustness and represents the main obstacle to overcome to develop therapeutic strategies. It is then essential to establish successful assays enabling phenotypic, (epi)genetic and functional identification and characterization of tumor subpopulations driving the tumorigenesis and delineating clinically relevant targets.

Many cancers (i.e. leukemia, breast, colon, brain, prostate, melanoma) depend for their continued growth and propagation on a population of cells called cancer stem-cells (CSCs) or tumor-initiating cells (TICs): these cells are slow-dividing, endowed with unlimited proliferation capacity, functionally defined by their tumorigenic capability when engrafted in mice, unresponsive to standard treatments, thus highly competent in repopulating the tumor [4–11].

TICs appear to be relatively rare in most human cancers, ranging from 0,0001% to 0,1% of the bulk tumor cell population [5], [6], [12–15]. However, recent studies report that TICs are frequent, indicating frequencies ranging from 25% to 40% [8, 16–18].

Of note, different aspects may influence TIC estimate: *in vitro* cell manipulation before transplantation, the use of agents (i.e. matrigel) sustaining tumor cell transplantation, the extent of the immunodeficiency of the recipient host, the duration of the experimental period for tumor formation following tumor cell injection and the experimental procedure implemented for TIC isolation. With this regard, although the exact contribution of each cell-surface marker in identifying the TICs is puzzling and unclear, current protocols are still based on the expression of putative stem-cell markers that could distinguish a small subpopulation of cells with tumorigenic potential from the majority of non-tumorigenic cells. In addition, the gold standard to determine TIC frequency within a tumor is the limiting dilution cell transplantation assay (LDA) [19]. Nevertheless, despite several studies regarding TIC frequencies, only rarely tumoral cells are transplanted in limiting dilution experiments.

The variability in TIC frequencies assessment challenges the prognostic role of TICs in predicting the clinical outcome in cancer patients. Thus far, the prognostic value of TICs has been linked in several types of tumors to the presence of stem-related features, such as the expression of stem-cell markers, genetic features, and tumorsphere formation [20–22]. Similar evidences have been reported also in GBM, where stem-cell marker expression (i.e. CD133, nestin) [23, 24] and neurosphere formation [25] have been associated to clinical outcomes. However, data from different groups are controversial, since GBM stem-cell marker expression is not always associated to a prognostic significance [26].

Here, we specifically estimate GBM TIC frequency employing limiting dilution transplantation of cells isolated from freshly-dissociated human GBMs. We believe that, owing the lack of definitive markers, only functional criteria applied on freshly-dissociated human GBMs will allow an unbiased assessment of the TIC content within parental GBMs. Furthermore, through *in vitro* neurosphere assay we look for the existence of any correlation between the sphere-forming capability and the *in vivo* tumorigenic potential of cells from the same human sample. Moreover, we investigate the effect of *in vitro* culturing primary GBM cells as neurospheres on their TIC content.

## RESULTS

### TICs from freshly-dissociated GBMs are rare

**Figure 1:**
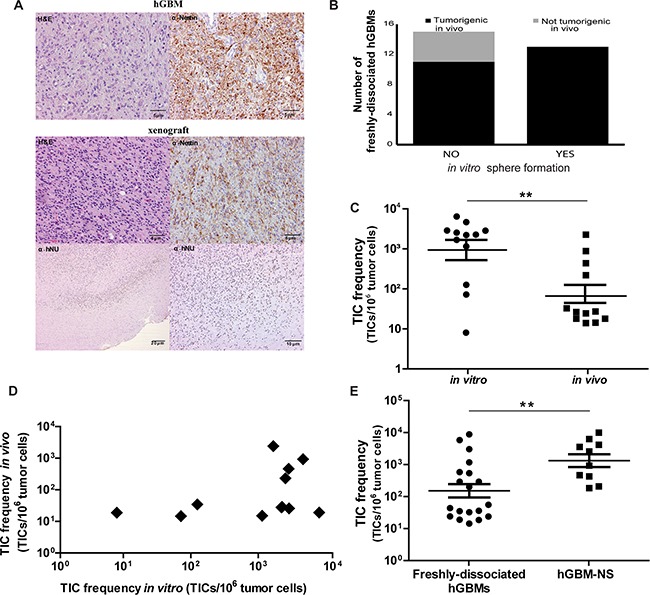
Dissociation between sphere-forming capacity and tumorigenic ability of freshly-dissociated hGBMs **A.** IHC analysis of a representative human GBM and its correspondent xenograft. The upper part of the panel depicts H&E and Nestin antibody staining of human glioblastoma; in the lower part, H&E and Nestin antibody staining of its correspondent xenograft are shown. Human anti-nuclei (α–hNU) antibody staining of the xenograft demonstrates the human origin of the tumor. Scale bars = 5 μm (H&E and Nestin), 20 μm and 10 μm (hNu). **B.** The graph represents the *in vitro* sphere formation ability and the *in vivo* tumorigenicity capacity of freshly-dissociated hGBMs: the 86% (n=24) of the specimens analysed are tumorigenic *in vivo* and the 54% (n=13) of these are able to give rise to spheres when placed in cultured. The remaining 11 specimens (46%) present an uncoupled sphere-forming capacity and tumorigenic ability, since they do not succeed to generate *in vitro* neurospheres. **C.** Comparison of TIC frequencies in freshly-dissociated hGBMs of 12 matched samples calculated through *in vitro* methylcellulose assay (mean 0.00093; C.I. 0.00026÷0.00334 after reverse logarithmic transformation) and *in vivo* limiting dilution assay (mean 0.00007; C.I. 0.00002÷0.00022 after reverse logarithmic transformation). The *in vitro* TIC frequency results to be 10-fold higher than the *in vivo* frequency (Paired samples *t*-test: t=4.233; df=11; P=0.0014**). **D.** Representation of the absence of correlation between TIC frequencies assessed through *in vivo* and *in vitro* assays of the 12 matched samples illustrated in Figure 1C (Pearson correlation after logarithmic transformation with r=0.391; P=0.209^ns^; n=12). **E.** Comparison of TIC frequencies assessed in freshly dissociated hGBMs (mean 0.00012; C.I. 0.00004÷0.00033 after reverse logarithmic transformation; n=19) and in hGBM-NS (mean 0.00105; C.I. 0.00037÷0.00298 after reverse logarithmic transformation; n=10) through *in vivo* LDA: TIC content in hGBM-NS was remarkably higher (Unpaired Student *t*-test: t=2.935, df=27, P=0.0067**). All statistical tests are two-sided.

Immediately after surgical removal, GBM specimens (n=28) were enzymatically and mechanically dissociated and viable cells immediately injected in the mouse brain without any *in vitro* manipulation to evaluate the ability to generate tumors. In parallel, cells were also plated in non-adherent serum free stem-cell medium to allow neurosphere formation. The 86% of the specimens analysed (n=24) was tumorigenic *in vivo* (Table 1), giving rise to tumors phenotypically similar to the parental ones (Figure 1A). Of these, the 54% (n=13) were able to give rise to spheres *in vitro* while the remaining 46% (n=11) did not (Table 1 and Figure 1B). Interestingly, xenografted cells from GBMs with uncoupled sphere-forming capacity and tumorigenic ability, once isolated always formed GBM in secondary xenotransplantation albeit still failed to generate neurospheres *in vitro* (data not shown). Thus, the inability to grow in culture cannot predict *in vivo* tumorigenicity.

**Table 1:** Clinical and experimental data of collected GBM patients

Clinical Data								MRI			Therapy		Experimental Data	
Patient ID	Age	Sex	Initial KPS	PFS	OS	FU	Status	Tumor size	C/L inv	Invas	Rad Surg	CCRT	Neurospheres formation	Tumor formation *in vivo*
GBM#76	55	M	60	2	2	30	D	82×43 mm	N	1	N	N	N	N
GBM#79	78	M	50	nd	nd	30	nd	40×50 mm	N	2	Y	nd	N	Y
GBM#89	71	M	70	5	12	26	D	34×23 mm	N	3	N	Y	N	N
GBM#92	66	F	90	20	20	20	A	23×21 mm	N	3	N	Y	Y	Y
GBM#93	59	F	70	nd	nd	20	nd	34×34 mm	N	1	Y	nd	Y	Y
GBM#94	51	F	90	4	17	20	D	54×40 mm	N	1	Y	Y	N	Y
GBM#98	52	M	90	17	20	20	A	25×13 mm	N	3	Y	N	Y	Y
GBM#99	65	M	80	nd	nd	19	nd	81×50 mm	Y	2	N	nd	N	Y
GBM#101	52	F	70	5	13	19	D	51×25 mm	N	2	Y	Y	N	Y
GBM#103	63	F	80	19	19	19	A	50×32mm	N	2	Y	Y	N	N
GBM#106	48	M	80	nd	nd	18	nd	52×45 mm	N	3	Y	nd	N	Y
GBM#107	72	M	60	nd	3	18	D	87×44 mm	N	1	N	Y	Y	Y
GBM#109	59	F	90	nd	nd	17	nd	50×43 mm	N	2	Y	nd	N	Y
GBM#110	53	M	90	7	17	17	A	30×36mm	N	2	N	Y	N	Y
GBM#115	57	F	90	nd	nd	16	nd	59×54 mm	Y	1	N	nd	Y	Y
GBM#116	51	M	90	nd	nd	16	nd	multicentric	N	multi centric	N	nd	N	Y
GBM#119	80	M	70	3	15	15	A	39×37 mm	N	2	Y	Y	Y	Y
GBM#121	66	M	80	15	15	15	A	48×42 mm	N	3	Y	N	N	N
GBM#122	42	F	90	3	15	15	A	30×16 mm	N	1	Y	Y	Y	Y
GBM#124	56	F	90	2	4	15	D	multicentric	N	multi centric	N	Y	Y	Y
GBM#125	62	M	90	8	14	14	A	62×31 mm	N	2	Y	Y	N	Y
GBM#128	56	M	80	5	8	13	D	62×42 mm	Y	1	Y	Y	Y	Y
GBM#130	40	M	90	10	12	12	A	41×23 mm	N	3	Y	Y	Y	Y
GBM#132	52	M	90	11	11	11	A	35 x31 mm	N	1	Y	Y	Y	Y
GBM#133	54	F	90	6	11	11	A	47×21 mm	N	1	Y	Y	Y	Y
GBM#138	54	F	90	11	11	11	A	41×34 mm	N	1	Y	Y	Y	Y
GBM#139	79	M	80	3	6	11	D	72×52 mm	N	3	Y	N	N	Y
GBM#142	42	M	90	4	10	10	A	39×37 mm	N	2	Y	N	N	Y

Surgical specimens together with clinical records were collected from 28 consenting patients after surgery. Pathologists classified tumors as primary GBM. Overall patients enrolled in the study were 60% men and 40% women, with a median age of 58 years (range, 40-80 years) and a mean Karnofsky performance score of 80 (range 50-90). KPS: Karnofsky performance status; PFS: progression free survival; OS: overall survival; FU: follow-up; Status: D= dead, A= alive; nd= data not available; C/L inv: contralateral invasion; Invas: invasiveness of the tumor mass where 1 = distance of invasion < 2 × diameter of tumor mass; 2 = 2 × diameter of tumor mass < distance of invasion < 3 × diameter of tumor mass; 3 = 3 × diameter of tumor mass < distance of invasion; Rad Surg: radical surgery (Y= patients that underwent radicl surgery; N= patients that did not undergo radical surgery); CCRT: concurrent chemoradiotherapy. In the “experimental data” section are reported the success (Y=Yes) or failure (N=No) of neurospheres formation and *in vivo* tumor development of each collected sample.

Considering LDA as the gold standard to quantify the TIC content, we stereotaxically injected serial concentrations of freshly-dissociated hGBM cells ranging from 10^5^ to 10 cells [10]. LDA was carried to completion in 19 out of 24 GBMs (Table 1). We did not succeed in assessing the TIC frequency of four specimens (hGBM#92, hGBM#93, hGBM#94, hGBM#101, hGBM#107) due to the paucity of available cells. Tumor formation was monitored for nearly one year as a consequence of the long latency requested for tumor to arise at the lower cellular doses. TIC frequencies in freshly-dissociated hGBMs were highly variable between patients, ranging from 1/143 to 1/87599 (Table 2). On average, freshly-dissociated hGBMs contained rare TICs, as observed in other tumors [15]. Interestingly, also freshly-dissociated hGBMs unable to form neurospheres *in vitro* (n=9) comprised a rare, highly variable number of TICs (Table 1 and 2).

**Table 2:** TIC frequencies in freshly-dissociated hGBMs

Freshly-dissociated hGBMs		Number of Transplanted Cells				TIC Frequency^−1^
						(95% CI)
		10^5^	10^4^	10^3^	10^2^	Estimate
hGBM#79	Incidence	3/3	3/3	3/3	1/3	**216**
	Median Survival	66±8	60±2	103±0	168±51	(49-962)
hGBM#98	Incidence	1/2	2/3	0/3		**52571**
	Median Survival	334±0	319±0	377±0		(12418-222619)
hGBM#99	Incidence	2/2	1/3	3/3		**6297**
	Median Survival	122±3	215±107	155±23		(1769-22420)
hGBM#106	Incidence	3/3	3/3	3/3	1/2	**143**
	Median Survival	145±100	343±1	277±15	370±25	(22-918)
hGBM#109	Incidence	2/3	1/3	0/2	0/3	**67816**
	Median Survival	106±0	108±4	289±123	195±71	(19039-241569)
hGBM#110	Incidence	1/3	2/3	0/2	0/3	**87599**
	Median Survival	185±69	255±97	318±62	354±42	(23252-330030)
hGBM#115	Incidence	3/3	3/3	1/3		**2164**
	Median Survival	179±5	192±10	195±10		(487-91614)
hGBM#116	Incidence	1/2	2/3	0/3	0/3	**52680**
	Median Survival	314±13	301±48	221±0	356±0	(12466-222628)
hGBM#119	Incidence	3/3	3/3	3/3	0/3	**417**
	Median Survival	179±12	167±6	203±10	324±21	(117-1494)
hGBM#122	Incidence	2/3	3/3	0/2	0/2	**34826**
	Median Survival	59±5	59±11	212±158	227±137	(9539-127156)
hGBM#124	Incidence	2/3	2/3	2/4		**28969**
	Median Survival	138±32	260±75	161±24		(7664-109506)
hGBM#125	Incidence	1/3	1/3	1/3	1/3	**66380**
	Median Survival	164±15	288±68	270±6	310±28	(18769-234769)
hGBM#128	Incidence	2/3	0/3	1/3	2/3	**38765**
	Median Survival	59±24	196±138	87±104	196±108	(10939-137374)
hGBM#130	Incidence	3/3	3/3	2/3	0/3	**1072**
	Median Survival	188±10	175±20	201±15	295±29	(267-4312)
hGBM#132	Incidence	2/3	2/3	1/2	0/3	**36019**
	Median Survival	73±2	90±2	112±0	158±52	(9951-130386)
hGBM#133	Incidence	3/3	3/3	0/3	0/3	**4326**
	Median Survival	120±8	139±0	216±0	186±5	(1237-15139)
hGBM#138	Incidence	2/3	3/3	2/3	0/3	**22741**
	Median Survival	110±0	117±13	147±22	120±59	(5519-93718)
hGBM#139	Incidence	3/3	3/3	0/3	0/3	**4326**
	Median Survival	170±130	244±1	163±142	315±14	(1237-15139)
hGBM#142	Incidence	3/3	3/3	1/3	0/3	**2340**
	Median Survival	290±5	268±15	223±62	234±0	(571-9604)

The TIC frequency is calculated through limiting dilution transplantation of n=19 freshly-dissociated hGBMs. Data show the TIC frequency per transplanted cells (estimate, in bold) by means of the extreme limiting dilution analysis function assessed by ELDA algorithm (http://bioinf.wehi.edu.au/software/elda/). In the table, the tumor incidence and the survival time (in days; mean±SD) of tumor-bearing mice after injection of different concentrations of cells are also indicated.

### *In vitro* culture overestimates TIC content

Next, we assessed TIC frequencies *in vitro* by culturing cells derived from the same freshly-dissociated hGBM directly into methylcellulose-containing medium, which is able to sustain the growth of cells endowed with stemness characteristics [11, 12]. On average, *in vitro* TIC frequency (mean=0.00093; C.I.=0.00026÷0.00334; n=12) was 10-fold higher than *in vivo* frequency (mean=0.00007; C.I.=0.00002÷0.00022; n=12) (Paired samples *t*-test: P=0.0014**) (Figure 1C). Furthermore, *in vitro* TIC amount is not portending the extent of *in vivo* TIC frequency (Pearson correlation with r=0.08156; P=0.8011^ns^; n=12) (Figure 1D).

We next asked whether TIC frequencies might be modified culturing primary hGBM cells in non-adherent serum free stem-cell medium. It has been demonstrated that TICs propagated as neurospheres closely mimic the genotype, gene expression profile, and biology of the parental GBM from which they derive [27]. Thus, we propagated cells from additional independent freshly-dissociated hGBMs as neurospheres (hGBM-NS), and performed LDA. hGBM-NS TIC content (mean=0.00105; C.I.=0.00037÷0.00298; n=10) was higher than TIC frequencies assessed in freshly-dissociated hGBMs (mean=0.00012; C.I.=0.00004÷0.00033; n=19) (Unpaired *t*-test: P=0.0067**) (Figure 1E), fluctuating from 1/100 to 1/6819 (Table 3).

**Table 3:** TIC frequencies in hGBM-NS

hGBM-NS		Number of Transplanted Cells					TIC Frequency^−1^ (95% CI)
		10^5^	10^4^	10^3^	10^2^	10	Estimate
hGBM#7	Incidence	14/14	5/5	6/7	4/9	0/9	**352**
	Median Survival	47±7	52±8	71±11	61±9	0±0	(158-786)
hGBM#8	Incidence	6/6	8/8	7/7	6/11	2/4	**100**
	Median Survival	78±10	85±14	112±5	141±14	156±4	(47-206)
hGBM#9	Incidence	5/5	4/4	3/4	2/4	2/4	**303**
	Median Survival	93±0	154±33	184±39	196±72	243±16	(96-955)
hGBM#10	Incidence	3/3	3/3	2/3	1/3	1/3	**488**
	Median Survival	153±12	174±19	186±29	225±0	165±0	(141-1697)
hGBM#18	Incidence	4/4	6/6	5/6	4/6	3/7	**201**
	Median Survival	90±48	80±21	88±15	112±8	114±9	(73-558)
hGBM#20	Incidence		1/2	2/2	1/2	1/2	**2712**
	Median Survival		291±0	309±104	217±0	181±0	(514-14315)
hGBM#25	Incidence	2/2	3/4	3/4	1/4	0/4	**2942**
	Median Survival	142±0	122±25	149±25	191±0	0±0	(929-9327)
hGBM#27	Incidence		2/4	1/3	1/5	1/5	**6070**
	Median Survival		267±34	59±0	225±0	136±0	(2007-18370)
hGBM#154	Incidence		2/3	1/3	0/3	0/3	**6819**
	Median Survival		191±1	162±0	0±0	0±0	(1921-24218)
hGBM#155	Incidence		3/3	1/3	1/3	0/3	**1350**
	Median Survival		89±18	20±0	92±0	0±0	(318-5735)

The TIC frequency is calculated through limiting dilution transplantation of n=10 hGBM-NS. Data show the TIC frequency per transplanted cells (estimate, in bold) by means of the extreme limiting dilution analysis function assessed by ELDA algorithm (http://bioinf.wehi.edu.au/software/elda/). In the table, the tumor incidence and the survival time (in days; mean±SD) of tumor-bearing mice after injection of different concentrations of cells are also indicated.

### TIC number positively correlates with tumor incidence and inversely correlates with survival in mouse xenografts

**Figure 2:**
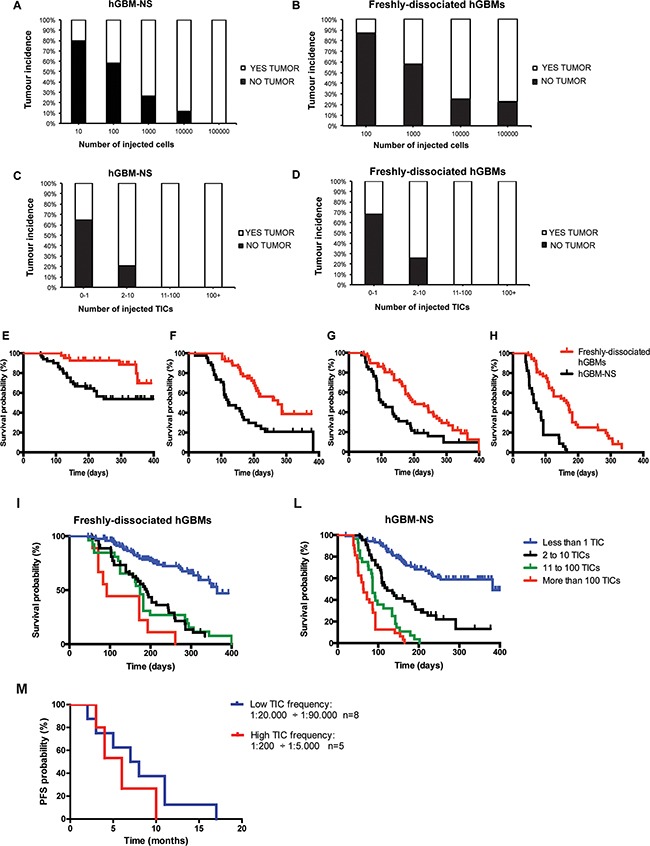
Tumor incidences and survival of mice intracerebrally injected with freshly-dissociated hGBM cells and cells from hGBM-NS The graphs depict the percentage of mice that developed tumours after orthotopic injection of 10, 100, 1000, 10000, 100000 cells from hGBM-NS cultures **A.** and freshly-dissociated hGBM cells **B.** In both conditions, we observe an increment of tumor incidence increasing the number of injected cells: at the maximum cell concentration, only the mice transplanted with cells from hGBM-NS achieve incidence of 100%. (Comparison A-B: G^2^ Wilks test where G^2^ =35.14; df=4; P<0.0001**). **C and D.** Correlation between tumor incidence and number of inferred TICs from hGBM-NS **(C)** and from freshly-dissociated hGBMs **(D)** The analysis reveals that tumor incidence is 100% both in hGBM-NS and in freshly-dissociated hGBMs where at least 11 TICs were injected (Comparison C-D: G^2^ Wilks test where G^2^ =0.58; df=4; P=0.97^ns^). **E.** and **H.** Kaplan–Meier analysis comparing mice injected with freshly-dissociated hGBM cells and cells from hGBM-NS cultures for (E) 100 cells (P=0.0027** by Log-rank test), (F) 1000 cells (P=0.0002** by Log-rank test), (G) 10000 cells (P=0.0022** by Log-rank test), and (H) 100000 cells (P<0.0001** by Log-rank test). **I.** and **L.** Kaplan–Meier analysis based on the number of TICs injected using freshly-dissociated hGBM cells (P<0.0001** by Log-rank test; n=9 to 120) (I) and cells from hGBM-NS cultures (P<0,0001** by Log-rank test; n=28 to 105) (L) shows a statistically significant advantage on survival when few TICs are injected. **M.** Kaplan-Meier analysis for progression free survival of a cohort of 13 GBM patients in relation to high TIC frequency (1:200-1:5000; n=5) and low TIC frequency (1:20000-1:90000; n=8). GBM patients with low TIC frequency experiences a longer, although not statistically significant, progression-free survival (median 7.5 months vs 6 months, P=0.33^ns^ by Log-rank test). Difference between groups is evaluated by means of the Log-rank test.

By pooling together the *in vivo* experiments performed with either freshly-dissociated hGBMs and hGBM-NS, we analysed the relationship between the total number of cells intracerebrally injected in immunocompromised mice and tumor incidence. In both conditions, we observed an increment of tumor incidence increasing the number of injected cells: at the maximum cell concentration (10^5^ cells), 100% of incidence was measured only with hGBM-NS (Figure 2A), in comparison to near 80% of incidence with freshly-dissociated hGBMs (Figure 2B) (comparison of tumor incidence reported in Figure 2A and 2B: G^2^ Wilks test where G^2^=35.14; df=4; P<0.0001**). The difference may be due to the smaller proportion of TICs within the freshly-dissociated hGBMs compared to hGBM-NS (Figure 1E). We thus proceeded analysing the relationship between the number of inferred TICs of each specimen and the corresponding tumor incidence. Interestingly, we obtained mirrored result in both hGBM-NS and freshly-dissociated hGBMs: up to 10 injected TICs, the incidence of the tumors increased with the number of injected TICs, while the injection of more than 10 TICs resulted in 100% of tumor incidence (comparison Figure 2C and 2D: G^2^ Wilks test where G^2^=0.58; df=4; P=0.97^ns^). Thus, TICs contained in freshly-dissociated hGBMs and hGBM-NS and extrapolated through *in vivo* LDA have the same tumorigenic potential.

Next, we compared the Kaplan-Meier plots obtained through the injection of the same amount of cells (10^2^, 10^3^, 10^4^, 10^5^ cells) derived from hGBM-NS and freshly-dissociated hGBMs. The survival curve of hGBM-NS was significantly shorter (Figures 2E and 2H), given the higher TIC frequency of hGBM-NS (1:2136) compared to that of freshly-dissociated tumors (1:26825) (for 10^2^ cells injected n=45^+^ per group, median survival undefined, P=0.0027** by Log-rank test; for 10^3^ cells injected n=43^+^ per group, median hGBM-NS survival=122 days and freshly-dissociated hGBMs survival=274 days, P=0.0002** by Log-rank test; for 10^4^ cells injected n=2^+^ per group, median hGBM-NS survival=101.5 days and freshly-dissociated hGBMs survival=203 days, P=0.0022** by Log-rank test; for 10^5^ cells injected n=34^+^ per group, median hGBM-NS survival=68 days and freshly-dissociated hGBMs survival=164 days, P<0.0001** by Log-rank test). Notably, when either freshly-dissociated hGBMs (Figure 2I) and hGBM-NS (Figure 2L) were stratified according to their TIC content, fewer was the amount of intracerebrally engrafted TICs, greater was mice survival (freshly-dissociated hGBMs: n=9 to 120, P<0.0001** by Log-rank test; hGBM-NS: n=28 to 105, P<0,0001** by Log-rank test). Similar results were obtained by stratification of GBM patients according to their TIC content. Given that TIC frequencies vary up to 500-fold between patients (Table 2), we divided GBM cohort in patients with low TIC content (1:20000-1:90000; n=8) and high TIC content (1:200-1:5000; n=5). Notably, patients with low TIC frequency experienced a longer, although not statistically significant, PFS (median 7.5 months versus 6 months, P=0.33^ns^ by Log-rank test (Figure 2M). Interestingly, putative stem-cell markers, markers associated with different GBM molecular subtypes [28, 29] and common GBM genetic alterations were similarly distributed between patients with low and high TIC content (Supplementary Figures S1 and S2; Supplementary Table S1A). Moreover, no association of these markers with neurosphere formation was found (Supplementary Table S1B).

## DISCUSSION

We established a quantitative assay that enables GBM TIC enumeration in human GBMs through direct injection of material immediately after the surgical procedure. Using this assay, we demonstrate that TICs are rare in GBMs. Moreover, TICs number is not uniform across patients and it is affected by *in vitro* culture manipulation, which overestimates the real TIC content.

Several research groups gave an estimation of GBM TIC frequencies, but these studies mostly relied on marker surface expression [12] (i.e. CD133 positive cells) or functional properties [9, 10, 30, 31]]. Such approaches often led to controversial results, since appropriate standardization methods to distinguish glioma stem-cells from progenitors or more differentiated cells are still lacking [32]. The estimated GBM TIC frequencies known in literature are mainly based on neurospheres [11, 12] and rarely on acutely dissociated tumors. Of note, the maintenance of surgical specimens for few passages, or even after one single passage, in a petri dish in serum-free condition can induce cell selection influencing the outcome of the frequency assessment [33]. In addition, a proper calculation of TIC frequency is still incomplete since tumor cells have been only rarely transplanted in cell dilution experiments.

We performed functional assays to determine the presence within freshly-dissociated human GBMs, of cells that more efficiently transplant the disease, and the occurrence of an analogy between TIC number calculated *in vivo* and *in vitro*. To address *in vivo* tumor propagation potential, we took advantage of an orthotopic xenograft model established in immunodeficient CD-1 nude mice [10, 34]. Different murine models have been developed to study human tumor xenografts, comprising severely compromised immunodeficient (SCID) mice, non-obese diabetic (NOD)-SCID or NSG mice [8, 15]. However, the use of more severely immunocompromised mouse strains could considerably increase TIC frequency, a scenario quite far from reality. Although the NOG mouse model shows markedly better engraftment of some tumoral cells than the NOD/SCID mouse [8, 15], according to our experience CD-1 mice survival was comparable to NOG survival.

We demonstrated that all GBMs analysed in this study contain rare TICs sustaining tumor growth, in accordance with other studies in GBMs and other types of tumors [5, 7, 12, 15, 17, 18]. Our results clearly show that not all human GBMs are able to grow *in vitro* but, remarkably, all of them are tumorigenic *in vivo*. *Ex-vivo* and *in vivo* experiments performed with cells kept in culture exclude from the analysis those GBMs not forming neurospheres, which we demonstrated are indeed representing a large proportion of tumors. Notably, the lack of correlation between tumor formation and ability to form neurospheres has been already demonstrated in a mouse model of glioma [35] and in a different cohort of GBM patients [36] emphasizing the importance of the cerebral compartment as a support for TIC growth and of *in vivo* limiting dilution assay on fresh specimens to assess the real TIC content.

However, controversial results exist demonstrating that *in vitro* sphere forming potential correlates with the *in vivo* tumorigenic potential in immunocompromised mice, as well as the ability to form neurospheres in culture can be considered a prognostic factor affecting GBM patient survival [25].

Performing a side-by-side analysis of the same specimen, we obtained an overestimation of TIC number when plating cells in semisolid substrate compared to xenotransplanting the same cells in immunocompromised mice. The higher TIC frequency *in vitro* is not surprising considering the known ability of progenitor cells to clone *in vitro* as well [10, 12]. In addition, the results make evident that the TIC frequency calculated through *in vitro* assays cannot portend the *in vivo* TIC frequency, reinforcing the need to investigate the tumor population immediately after surgical resection through *in vivo* LDA. Relevant is also the 10-fold difference quantified between the average TIC frequency of freshly-dissociated hGBMs and the average TIC frequency of *in vitro* maintained neurospheres. This different frequency might be explained by the composition of the two populations. Data from our group [10] and others' [9, 30] demonstrated, through the use of marker-independent methods, that neurospheres are mainly composed by slow-dividing cells endowed with stem-cells characteristics and high-dividing cells, tumorigenic and able to proliferate, although for only few rounds.

After the establishment of an accurate estimation of TIC number, we explored the TIC influence on tumor incidence and mice survival. Interestingly, few studies exist only in other types of tumors reporting a relation between TIC frequencies and tumor aggressiveness, implemented through *in vivo* LDA in immunodeficient recipient mice [15, 37]. In line with these studies, we found a positive association between TIC frequency and GBM incidence and we defined a threshold over 10 TICs sufficient to induce tumors in 100% of cases. In addition, TIC number inversely correlated with mice survival. Notably, GBM patients with low TIC frequency experienced a trend towards a longer progression free survival. However, we did not find any association between the clinical features (tumor size and tumor invasiveness) and TIC content. Despite the controversial results related to markers clearly describing the TIC and their relation with GBM patients' outcome [23, 24, 26], the expression of either putative stem-cell markers or markers associated with different GBM molecular subtypes [28, 29], or common genetic GBM hallmarks did not associate with either TIC content or neurosphere formation.

TICs derived from human GBMs have been characterized mainly in *in vitro* experiments and their effectiveness as informative tools in reflecting GBM pathophysiology, determining differentially regulated pathways as well as in exploring the potential efficacy of anticancer drugs is well recognized. With this study we demonstrate that *in vivo* examination of cells from freshly-dissociated GBMs in a context that closely resembles the original setting will provide an unbiased tool to analyse the widest range of GBMs. In addition, we believe that the assessed TIC frequency mirrors the real TIC content in human GBMs, especially in light of the recent findings related to the possible conversion of non-TICs to a TIC phenotype, which is dependent on microenvironmental cues and that can not be properly simulated in *in vitro* or *ex vivo* conditions [38–41].

Our findings establish the accuracy of TIC detection by injecting freshly-dissociated GBM cells, and the need for care when using *in vitro* cultured neurospheres. The orthotopic injection of acutely-dissociated human GBMs represents an unbiased pre-clinical tool for basic and translational research.

## MATERIALS AND METHODS

### GBM patients

Surgical specimens and clinical records were collected from 28 consenting patients in the Department of Neurosurgery at Neurological Institute “C. Besta” (Italy) under “C. Besta” research ethics committee approval. The specimens were analysed by pathologists and classified as primary GBM (WHO IV). Tumors displayed characteristics consistent with those reported in the literature concerning age, sex distribution, dimensional range, Karnofsky performance status scale (KPS) and invasiveness [36].

Overall survival and progression-free survival have been calculated as elapsed time from surgery to death or from surgery to the diagnosis of recurrence/progression. The patients enrolled in the study were 60% men and 40% women, with a median age of 58 years (range, 40-80 years) and a mean Karnofsky performance score of 80 (range 50-90). The mean follow up was 17 months (range 10-30); 7 patients were lost to follow up, whereas for the others mean progression free survival (PFS) was 8+/−5.8 months, while overall survival (OS) was 11.8+/−5.1 months, in accordance to RANO criteria [42]. Tumor invasiveness has been calculated as well as was evaluated invasiveness in the contralateral hemispheres. Distance of infiltration was estimated measuring the oedema area in FLAIR sequences in MRI. The majority of selected patients received radical surgery and concurrent chemoradiotherapy (CCRT).

### Tumor sample preparation and *in vitro* assay

TICs were isolated from GBM surgical specimens as previously described [10]. Briefly, fresh tumors were finely minced, enzymatically digested with papain (2 mg/ml; Worthington Biochemical, Lakewood, NJ) at 37°C and mechanically dissociated until achievement of single cell suspension. To remove red blood cells, the single cell suspension was incubated at room temperature for 3 – 5 minutes with ACK (Ammonium-Chloride-Potassium) Lysing Buffer and then separated from debris using Percoll density gradient centrifugation. Viable cells were resuspended in serum-free medium (Dulbecco's modified Eagle medium/Ham's F12 Nutrient Mixture; StemCell Technologies) supplemented with B27 supplement (Life Technologies, Paisley, United Kingdom; www.invitrogen.com), 20 ng/ml epidermal growth factor, 10 ng/ml basic fibroblast growth factor (PeproTech, Rocky Hill, NJ). Human GBM neurospheres were grown as spheroid aggregates as previously described [10].

To evaluate the capacity to form neurospheres, cells were resuspended in Dulbecco's modified Eagle medium/F12 medium containing methylcellulose (StemCell Technologies, Vancouver, BC, Canada) and seeded on a minimum of three 35 mm culture plates (3000 cells/dish). Two weeks after plating, the number of clones was counted. The ratio between neurospheres formed and number of single cells plated corresponds to the percentage of TICs in the plate.

### In Vivo Limiting Dilution Transplantation Assay

Decreasing cell concentrations (10^5^-10 cells) derived from both freshly-dissociated GBM cells and hGBM-NS were resuspended in 2 μl of phosphate-buffered saline (PBS) and stereotaxically injected into the nucleus caudatus (coordinates from bregma: 1 mm posterior, 3 mm left lateral, and 3.5 mm in depth) of 5 weeks old female nu/nu CD1 mice (Charles River, Wilmington, MA; http://www.criver.com). Mice were intraperitoneally anesthetized with tribromoethanol (0.1 ml/10 g of body weight). The experiments were performed in accordance with the Italian laws (D.L.vo 116/92 and following additions), which enforce EU 86/609 Directive (Council Directive 86/609/EEC of 24 November 1986 on the approximation of laws, regulations and administrative provisions of the Member States regarding the protection of animals used for experimental and other scientific purposes). The mice were maintained until development of neurologic signs, and the brains of killed mice were collected.

### Statistical Analysis

Paired samples *t*-test was used to compare TIC frequency calculated through *in vitro* methylcellulose assay and *in vivo* LDA on matched freshly-dissociated hGBMs. TIC frequency assessed through *in vivo* LDA in freshly-dissociated hGBMs and in hGBM-NS was compared performing the unpaired Student *t*-test. The relation between matched values of *in vivo*/*in vitro* TIC frequency was evaluated by means of correlation analysis. For the *in vivo* LDA, TIC frequency and statistical significance were estimated by means of the extreme limiting dilution analysis function (http://bioinf.wehi.edu.au/software/elda/). The incidences of tumors per number of injected cells and injected TICs were compared by means of Log-linear analysis (G^2^ test; http://www.biostathandbook.com/gtestgof.html). In Kaplan–Meier curves, survival differences were compared by Log-rank test. P-values less than 0.05 were considered statistically significant (**) unless otherwise indicated. All statistical tests were two-sided.

## SUPPLEMENTARY MATERIALS


