# Supplementary material for: Tumor-initiating cell frequency is relevant for glioblastoma aggressiveness
Source: Oncotarget. 2016 Aug 25;7(44):71491–503. doi: 10.18632/oncotarget.11600 (PMC5342095; doi:10.18632/oncotarget.11600)
Supplement: Supplementary file 1 [file oncotarget-07-71491-s001.pdf]

Tumor-initiating cell frequency is relevant for glioblastoma aggressiveness

SUPPLEMENTARY MATERIALS

Immunohistochemistry (IHC)

Patients' specimens were fixed in Carnoy's solution, while the correspondent xenografted mouse brains were formalin-fixed and then both dehydrated, paraffin-embedded and sectioned at 2 µm according to established procedures. For hematoxylin-eosin staining, slides were stained in Carazzi hematoxylin solution, rinsed in running tap water and counterstained in eosin solution. Antigen's retrieval was performed at pH 9 and 90°C in a PT Link pre-treatment module (Dako, Glostrup, Denmark) when requested. Slides were first blocked in 3% H_2O_2 (Sigma-Aldrich, St. Louise, Missouri, USA), then they were incubated with Normal Goat Serum (Dako, Glostrup, Denmark) and with the respective primary antibody: mouse monoclonal anti-EGFR (1:100, Neomarkers, Fremont, CA, USA), mouse monoclonal anti-PTEN (1:100, Santa Cruz Biotechnologies, Dallas, Texas, USA), mouse monoclonal anti-TP53 (1:50, Dako, Glostrup, Denmark), mouse monoclonal anti-MGMT (1:50, Millipore, Darmstadt, Germany), mouse monoclonal anti-IDH1 R132H (1:100, Dianova, Hamburg, Germany), mouse monoclonal anti-MIB1 (1:100, Dako, Glostrup, Denmark), rabbit polyclonal anti–activated-NOTCH1 (1:100, Ab8925, Abcam, Cambridge, UK), mouse monoclonal anti-Human Nuclei (1:1000, Millipore, Darmstadt, Germany), mouse monoclonal anti-Nestin (1:200, R&D Systems, Minneapolis, Minnesota, USA), rabbit polyclonal anti-Olig2 (1:200, Invitrogen, Life Technologies, Carlsbad, USA), goat polyclonal anti-YKL-40 (1:500, Santa Cruz Biotechnologies, Dallas, Texas, USA), rabbit polyclonal anti-Sox2 (1:50, Stem Cell Technologies, Vancouver, Canada), mouse monoclonal anti-GFAP (1:200, clone 6F2, Dako, Glostrup, Denmark), mouse monoclonal anti-CD15 (1:100, BD Pharmingen, San Josè, California, USA). Sections were subsequently incubated with anti-mouse or anti-rabbit Envision® peroxidase conjugated (Dako, Glostrup, Denmark) as secondary antibody, for 1 hour at room temperature. Finally, slides reacted with diaminobenzidine (DAB Substrate Chromogen System, Dako Cytomation, Glostrup, Denmark), counterstained with hematoxylin, mounted and visualized using a bright field microscope.

Statistical Analysis

We adopted Fisher's exact test to compare protein markers positivity frequency (number of occurrences) between GBMs forming neurospheres and GBMs not able to form neurospheres. Unpaired Student *t*-test with Bonferroni correction for multiple testing was adopted to compare percent TIC frequency values between samples positive or negative for each marker. Statistical analysis was performed using Prismv6.0 software (GraphPad Software, La Jolla, CA). P-values less than 0.05 were considered statistically significant (**) unless otherwise indicated. All statistical tests were two-sided.

SUPPLEMENTARY FIGURES AND TABLE

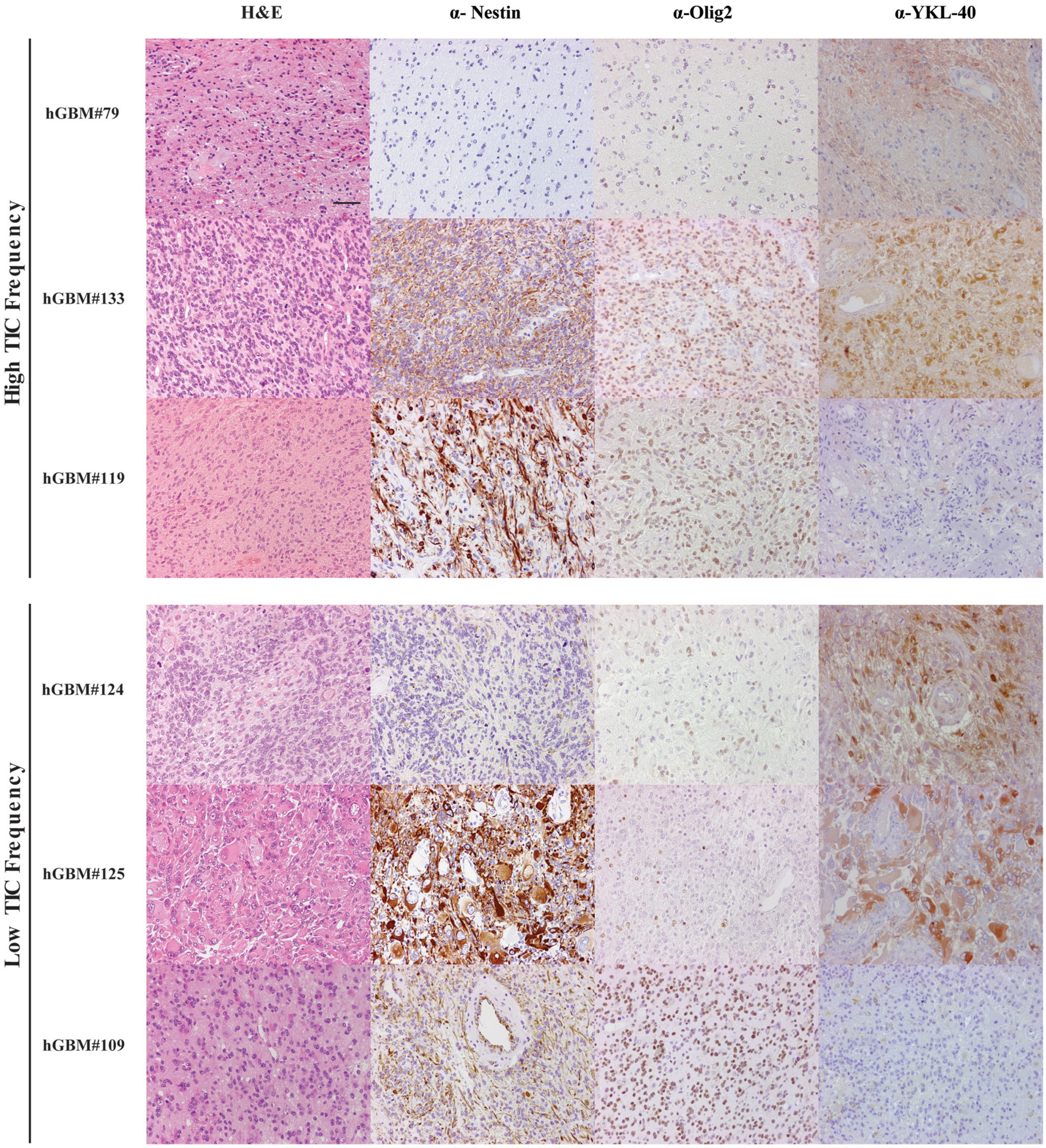

Supplementary Figure S1: Immunohistochemistry analysis of tumors derived from 3 patients with low TIC frequency and 3 patients with high TIC frequency. Representative images of H&E and tumor markers in 6 different patients displaying a different TIC content (hGBM#124, hGBM#125, hGBM#109 TICs < 1:20000 and hGBM#79, hGBM#133, hGBM#119 TICs >1:5000). The variable expression level of Nestin, Olig2, YKL-40 reveals that none of these markers is associated with the number of TICs. Scale bar = 5 μm.

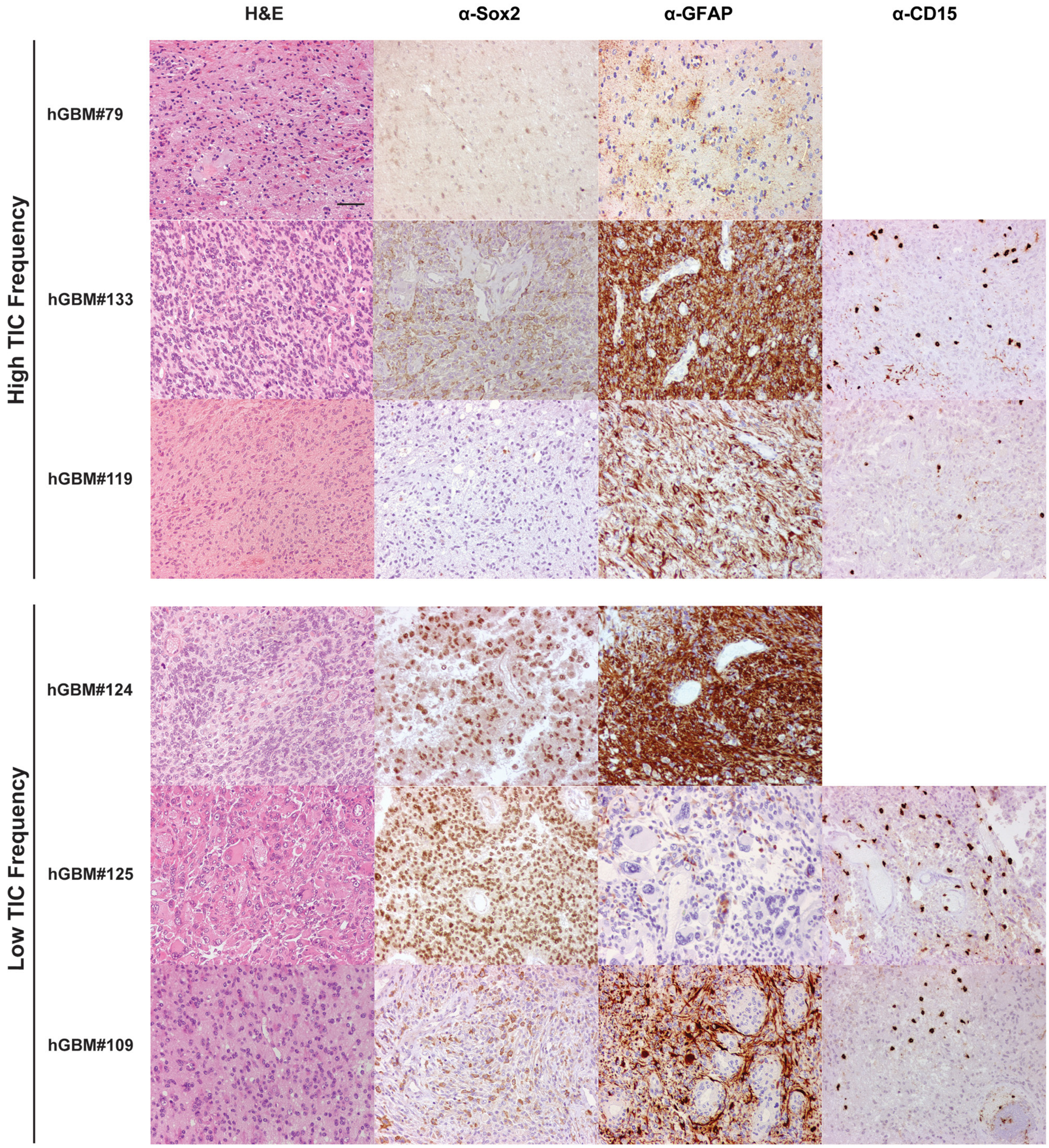

Supplementary Figure S2: Immunohistochemistry analysis of tumors derived from 3 patients with low TIC frequency and 3 patients with high TIC frequency. Representative images of H&E and Sox2, GFAP and CD15 immunostains are provided for 6 different patients displaying a different TIC content (hGBM#124, hGBM#125, hGBM#109 TICs < 1:20000 and hGBM#79, hGBM#133, hGBM#119 TICs >1:5000). The variable expression level reveals that none of these markers is associated with the number of TICs. Scale bar = 5 μm.

Supplementary Table S1A: Analysis of tumor marker expression and TIC content or neurospheres formation. A. Comparison of TIC frequency obtained by *in vivo* limiting dilution assay between tumors positive and negative for the indicated proteins (positivity threshold set at the 20% of positive cells, except for IDH1 where positivity indicates presence of the R132H mutation); average values are shown with Standard Error of the Mean. It was not possible to perform a Student t-test for those markers (IDH1 and MIB1) with just one case either positive or negative in one of the two classes. None of the markers resulted significantly different at the t-test after Bonferroni correction for multiple testing (maximum P-value for significance set at P=0.00625)

	Positives			Negatives				
	N	TIC frequency ($\times 10^{-4}$)		N	TIC frequency ($\times 10^{-4}$)		Student *t*	P value
**MGMT**	6	10.10	± 7.38	13	8.18	± 5.45	0.204	0.841
**PTEN**	7	11.68	± 9.79	12	7.11	± 4.05	0.504	0.620
**P53**	9	8.24	± 5.42	10	9.28	± 6.80	0.118	0.907
**EGFR**	6	1.34	± 0.74	13	12.23	± 6.09	1.195	0.248
**YKL-40**	11	7.93	± 6.25	8	9.98	± 5.92	0.230	0.821
**Olig2**	13	2.98	± 0.18	6	21.38	± 1.21	2.199	0.042
**Notch1**	4	8.44	± 5.61	13	10.05	± 6.08	0.140	0.891
**Nestin**	7	3.25	± 2.35	10	8.90	± 6.36	0.948	0.358
**Sox2**	6	0.56	± 0.35	11	8.32	± 4.36	1.295	0.215
**GFAP**	11	3.13	± 2.13	6	10.07	± 7.39	1.146	0.270
**MIB1**	16	1.68	± 0.63	1	23.98		not performed	
**IDH1**	1	0.15		18	9.27	± 5.19	not performed	
**CD15**	5	5.39	± 4.67	1	46.30		not performed	

Supplementary Table S1B: Frequency of positive or negative tumors according to the indicated markers (positivity threshold set at the 20% of positive cells, except for IDH1 where positivity indicates presence of the R132H mutation) relatively to samples forming or not neurospheres *in vitro*. For each 2x2 contingency table the odds ratio, the positive predictive value (fraction of NS+ ["neurosphere forming" tumors] found positive for the marker), and the negative predictive value (fraction of NS- [not "neurosphere forming"] tumors found negative for the marker) are shown. None of the markers was found significant at the Fisher exact test

	NS+	NS-	Odds Ratio	Positive predictive value	Negative predictive value	P value (Fisher exact test)
**MGMT+**	3	8	0.375	0.273	0.5	0.276
**MGMT-**	10	10				
**PTEN+**	3	6	0.6	0.333	0.545	0.696
**PTEN-**	10	12				
**P53+**	6	9	0.857	0.4	0.563	1
**P53-**	7	9				
**EGFR+**	6	4	3	0.6	0.667	0.247
**EGFR-**	7	14				
**YKL-40+**	7	6	2.33	0.538	0.667	0.294
**YKL-40-**	6	12				
**Olig2+**	10	10	2.67	0.5	0.727	0.276
**Olig2-**	3	8				
**Notch1+**	6	4	3	0.6	0.667	0.243
**Notch1-**	6	12				
**Nestin+**	6	4	1.13	0.6	0.429	1
**Nestin-**	4	3				
**Sox2+**	3	7	0.571	0.3	0.571	0.644
**Sox2-**	3	4				
**GFAP+**	8	2	5.33	0.8	0.571	0.162
**GFAP-**	3	4				
**MIB1+**	12	13				not performed
**MIB1-**	1	1				
**IDH1+**	0	2				not performed
**IDH1-**	13	16				
**CD15+**	3	0				not performed
**CD15-**	2	1				
